# Corticosteroids treatment for pediatric acute respiratory syndrome: A critical review

**DOI:** 10.15537/smj.2023.44.5.20220672

**Published:** 2023-05

**Authors:** Khouloud A. Al-Sofyani

**Affiliations:** *From the Department of Pediatric, Pediatric Critical Care Unit, Faculty of Medicine, King Abdulaziz University, Jeddah, Kingdom of Saudi Arabia*

**Keywords:** PARDS, ARDS, steroid, PALICC

## Abstract

Approximately 25% of all pediatric consultations are due to respiratory conditions, 10% of which are for asthma. Regarding prevalence, bronchiolitis, acute bronchitis, and respiratory infections are other leading pediatric respiratory illnesses. Compared to the aforementioned diseases, pediatric acute respiratory distress syndrome (PARDS) is rare but lethal in the Intensive Care Unit patients. According to global studies, the mortality in PARDS ranges from 13.3% to 60.7%. Before the Pediatric Acute Lung Injury Consensus Conference (PALICC), adult acute respiratory distress syndrome (ARDS) management guidelines were used for PARDS. The PALICC set new criteria to identify PARDS with a different treatment and management approach. Steroids have been used to treat ARDS in some cases, although their effectiveness in treating pediatric patients is highly debated in the scientific community. This review examines steroid use in treating PARDS, emphasizes current developments in the field, and gives a broad overview of PARDS management.

**P**ediatric acute respiratory distress syndrome (PARDS) is a rare disease from a general public health perspective and is less common than illnesses such as cancer or asthma. However, the resources utilized in its treatment and the higher percentage of danger it poses to the lives of patients make this disease worthy of extensive scientific investigation. Ashbaugh et al^1^ provided initial pathological description of acute respiratory distress syndrome (ARDS). Later, in 1976, Katzenstein et al^2^ identified its first distinctive histopathological feature, named diffuse alveolar damage (DAD). The ARDS was first described by the American European Consensus Conference (AECC) in 1994 in Barcelona, Spain.^3^ In an improvement to the Barcelona definition, a revised ARDS definition for adult patients was proposed in Berlin in 2012, focusing on the practicality, reliability, validity, and objective evaluation of the previous guidelines.^4^ This definition has been verified and applied to children with severe respiratory distress. In 2015, the Pediatric Acute Lung Injury Consensus Conference (PALICC) differentiated PARDS from ARDS for the first time; the conference established the need for a specific identity for PARDS.^5^ This conference was the first one to understand the characteristics of PARDS-specific syndrome. The PALICC established the new criteria to identify PARDS (Table 1).^6^ A study indicated that the PALICC criteria resulted in identification of more ARDS patients than by the Berlin definition. However, more severe cases were identified when both Berlin and PALICC criteria were used together rather than criteria one.^7^

**Table 1:** - Difference between different PARDS criterion.

Parameters	Previous criterion (1994)	Current criterion (2012)
Age	Includes patients of all ages	Pediatric patients (excludes individuals having perinatal lung disease)
Duration	Not specified	New known injury worsened within 7 days
Origin of edema	Pulmonary artery wedge pressure ≤17 mm Hg	Respiratory failure is not entirely explained by cardiac failure
Chest imaging	Bilateral infiltrate in radiography	New infiltrate with severe pulmonary parenchymal disease in radiography
Oxygenation	PaO_2_/FiO_2_: acute lung injury <300 mm Hg	PaO_2_/FiO_2_: mild: 200 to 300 mm Hg, moderate: 100 to 200 mm Hg, Severe: ≤100 mm Hg
Positive end-expiratory pressure	Not specified	≥5 cm H_2_O
Steroid treatment	Late-stage PARDS treated with a high dose of steroids after 12 days of mechanical ventilation	Steroid treatment is prohibited

PARDS: pediatric acute respiratory distress syndrome

The mortality rate of this disease ranges from 13% to 60.7%, as reported in different studies across the globe.^6^ A survey of 146059 patients, below 18 years of age, who were hospitalized to the Intensive Care Unit (ICU) with traumatic injury showed 20% morbidity. Moreover, even after adjusting for the severity of injury and hemodynamic anomalies, children who develop ARDS after a traumatic injury have higher morbidity and mortality risks. The PARDS associated with immunodeficiency have a substantially higher mortality rate (60.7%) when analyzed using the PALICC criteria.^8^ Over the past 10 years, the lack of improvements in outcomes has highlighted the demand for new therapeutic approaches and methods to prevent ARDS in children with traumatic injuries.^9^ The ARDS is divided into subtypes, allowing for a better understanding of its heterogeneity, resulting in discovery of potential target therapies.^10-13^

Similarly, PARDS subtypes were reported in a cohort of patients hospitalized in the Children’s Hospital of Philadelphia, United States. The reported PARDS subtypes were due to direct (pulmonary) and indirect (non-pulmonary) causes with different and distinct clinical features but with comparable results and mortality risks. On the other hand, the infectious or non-infectious PARDS subtype showed heterogeneity in the clinical features, mortality, and predictors of mortality.^14^ An important retrospective observational study on PARDS non-survivors at the Children’s Hospital of Philadelphia (CHLA) was performed by Dowell et al.^15^ Their study clearly stated that after the onset of PARDS, the median time of death was 6 days, with an overall mortality rate of 19%. A higher severity of PARDS is linked to early fatalities. The presence of comorbidities, such as immunodeficiency in patients with PARDS, accounted for 51% of deaths after more than 7 days and approximately 35% of all non-survivors.^15^

Additionally, according to the findings of Schouten et al,^16^ neither the incidence nor mortality of PARDS has changed during the past 20 years, and the mortality rate is highly correlated with the geographical location.^16^ According to Schouten et al,^16^ the population-based overall mortality rate of PARDS is 3.5 per 100000 people, while a 2.3% mortality was reported in pediatric intensive care unit (PICU)-admitted patients. This percentage could rise to 7%–10% of all children who receive mechanical ventilation. While systemic illness or (severe) trauma are frequent causes of extrapulmonary PARDS, infection due to virus or bacteria typically result in pulmonary ARDS in children. Geographically, Asian countries have registered a higher mortality rate of 51% among PARDS patients compared to 27% in Western countries. The PARDS Incidence and Epidemiology (PARDIE) study is one of the most extensively accessible epidemiological research to date. Data from 135 PICUs in 27 different countries were collected. According to the PARDIE study, 3% of patients receiving care in PICUs have PARDS, and the total mortality rate is 17%. In the serious cases of hypoxemia, the mortality rate in patients surpasses 30%. It is evident from these studies that PARDS treatment requires the development of effective therapies. Smaller airways, less firm chest walls, and lower functional residual capacity are only characteristics that distinguish pediatric patients’ lungs from those of adults.^17,18^ Most significantly, children’s lungs develop and grow until they reach their adult height, which makes the pathophysiological response of a child to infection and injury different from those of adults.^19^ The clinical manifestations of ARDS include tachypnea, dyspnea, hypoxemia, shortness of breath, rapid breathing, widespread crackles, and tachycardia.^20^

The management of PARDS in the PICU is performed by addressing the underlying cause, ensuring sufficient oxygenation, preventing further lung damage, and avoiding additional pulmonary problems.^18,21,22^ Children with moderate PARDS may be candidates for early noninvasive positive pressure ventilation (NPPV). While assisting in increasing tidal volumes and lowering breathing effort, continuous positive end-expiratory pressure (PEEP) helps expand airways, improve alveolar recruitment, and enhance oxygenation. For individuals who are suffering from severe hypoxemia, NPPV is not advised. There are also indications for intubation and invasive ventilation when there is evidence of increased oxygen demand and respiratory effort.^23,24^ High-frequency oscillatory ventilation (HFOV) and traditional mechanical ventilation have also been evaluated in randomized controlled studies with PARDS patients. The results showed that HFOV was better in increasing oxygenation in patients; however, it did not improve mortality outcomes in patients.^25^ The PALICC does not regularly advocate using iNO (inhaled nitric oxide) for PARDS. Nevertheless, it may be used as a last resort or a link to extracorporeal life support in patients who suffer from pulmonary hypertension or acute right ventricular failure.^26^ Surfactants are effective in managing ARDS in adult patients but are typically thought to be ineffective for treating ARDS in children.^26^

Prone positioning is used effectively in managing adult ARDS but is not advocated as a standard treatment in PARDS; however, in severe PARDS patients, prone positioning can be used as an option.^27^ Extracorporeal membrane oxygenation (ECMO) is recommended by the PALICC when alternative treatment methods fail to sustain sufficient gas exchange in patients suffering from severe PARDS.^26^ Ventilator-free days, decreased oxygenation, high mortality, and acute renal damage are linked to high cumulative fluid balance and increased mortality in PARDS patients; proper fluid management is essential.^28^ Because the critical disease is associated with a higher baseline metabolic rate and enhanced protein catabolism, ARDS patients are especially vulnerable to malnutrition. Malnutrition can cause muscular weakness, decline in respiratory and cardiac muscle function, and also results in the decline of lean body mass. Children with PARDS show higher survival rates when they consume sufficient protein and other nutrients.^29^ Mechanically ventilated children with ARDS frequently require sedatives or analgesics to help them synchronize with the ventilator and reduce their pain or anxiety. In a cluster-randomized trial performed on 2449 ARDS children, Curley et al^30^ found that sedation did not decrease the time required for mechanical breathing. Sedated patients experienced higher post-extubation stridor and longer days of intense pain and agitation. According to the PALICC recommendations, sedation should be carried out at a low but adequate level to enable successful ventilation. Since ARDS was first reported by Ashbaugh et al,^1^ steroids have been suggested as treatment and are frequently used. Their study revealed the use of steroids in 9 of 12 patients, showing a significant improvement in one patient, another had decent improvement, and no therapeutic benefit in other patients. Corticosteroids can reduce the severity of ARDS by suppressing the immunological and inflammatory systems. However, the application of corticosteroids in PARDS treatment is debatable.^26^

The present review examined and compared the existing evidence on steroid use in ARDS, with particular emphasis on PARDS. PubMed/Medline, Google Scholar, Picutrials.net, and ClinicalTrials.gov databases were used to conduct a literature search of papers written in English from the earliest date accessible to July 2022. We selected prospective randomized controlled trails (RCTs), meta-analyses, and observational studies for the analysis. The pathophysiology, pharmacological properties, and epidemiology were the characteristics used to differentiate between the adult and pediatric subgroups in this study.

## The rationale for steroid use in PARDS

**Figure 1:**
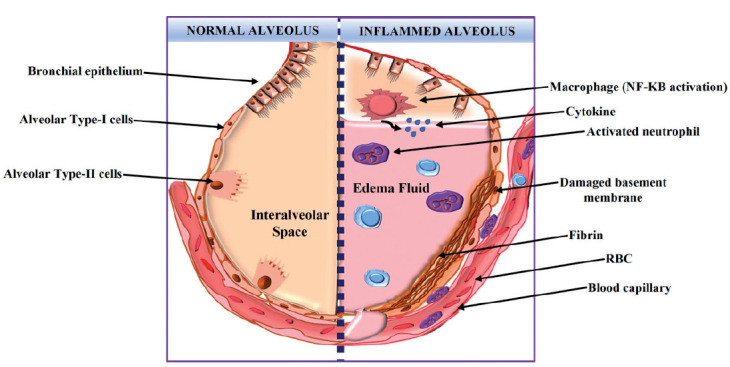
- A healthy alveolus with intact alveolar cell components and the vascular epithelial membrane is shown in the left panel. Following an acute inflammatory insult, alveolar alterations are seen in the right panel.

The ARDS is a clinical condition instead of being a particular disease entity that can be caused by many medical conditions, including sepsis, pneumonia, and trauma. The term primarily represents non-cardiogenic pulmonary edema with multiple etiologies, similar to many other disease conditions. Numerous insults have been shown to result in PARDS. Direct lung injury and indirect lung injury are 2 different causes that depend on whether the lungs are first harmed. The main causative factors of lung injury are pneumonia, inhalational lung injury, aspiration, lung contusion, chest injury, and submersion trauma. Sepsis, shock, pancreatitis, trauma, cardiopulmonary bypass, acute lung injury (ALI) associated with transfusions, burns, and raised intracranial pressure are indirect causes of PARDS.^31-34^ With an extensive alveolar injury, increased vascular permeability, and acute lung inflammation, ARDS causes reduced pulmonary compliance, poor gas exchange, and pulmonary hypertension, ultimately resulting in hypoxic respiratory failure Figure 1. Three stages can be distinguished in the pathogenesis of ARDS: the exudative phase, which is marked by the presence of activated resident alveolar macrophages and release of robust pro-inflammatory mediators and chemokines, causing interstitial and intra-alveolar flooding; the proliferative phase, when the provisional matrix is formed, the endothelial barrier function is restored; and the fibrotic stage, when interstitial and intra-alveolar fibrosis develops.^35^ In addition, several hyperinflammatory and cytokine release disorders result in secondary lung damage (CRS). The CRS is cytokine storm syndrome is severe and acute systemic inflammatory response, which is caused due to several factors, including infections and specific medications. An important underlying cause of CRS is hemophagocytic lymphohistiocytosis (HLH), a fatal condition brought on by severe excessive inflammation resulting from the multiplication of activated lymphocytes and macrophages and release of inflammatory cytokines. Seven out of 11 children with multi-organ failure who were diagnosed with HLH, showed signs of ARDS, according to Nahum et al.^36^

Immunity is activated in ARDS in 4 different ways. i) Innate immunity: Studies have confirmed the importance of innate immune system and associated cytokines to ARDS pathophysiology. Since ARDS was first described, neutrophils and innate immune system have been found to be linked to ARDS. Higher interleukin (IL)-6, IL-8, tumor necrosis factor (TNF), alveolar IL-6, TNF-, and IL-1 levels have been linked to elevated tidal volumes and pressure limits in two different experiments. Additionally, re-analyses of numerous ARDS network studies have shown the likelihood of 2 separate subgroups that are distinguished via inflammatory biomarkers, such as IL-6, IL-8, and TNF-receptor1.^37,38^ ii) Inflammasomes, damage-associated molecular patterns (DAMPs) can activate intracellular protein complexes called inflammasomes. The nucleotide-binding domain (NOD)-like receptor protein 3 (NLRP3) inflammasome studies have attracted maximum interest because it triggers caspase-1, which stimulates IL-1 and IL-18 expression. These cytokines were detected at increased levels in ARDS.^39^ The pharmacological inhibition of IL-1β has been shown to be a therapeutic agent in a mouse model. iii) Neutrophil extracellular traps (NETs): The extent of lung injury is proportional to a neutrophil influx in reaction to alveolar epithelial and macrophage signals. Peptidyl arginine deiminase 4 (PAD4)-mediated citrullination of histones controls NETs, a type of programmed neutrophil cell death. Toll-like receptor 4 (TLR4)-dependent signaling on platelets can initiate NETosis, and the ensuing extracellular chromatin lattices and antimicrobial agents capture pathogens. However, they can also lead to pulmonary damage and the production of more DAMPs. The PAD4 has become a possible therapeutic target because of its importance in NETosis.^40-44^ iv) T cells, adaptive immunity, and other inflammatory cells have also been linked to ARDS. Lately, researchers have investigated the role of CD4+ T-cells. The T-cell immune system has several distinct Th subsets, which include Th1, Th2, Th17, and regulatory T cells (Tregs). Tregs, an important T cell subtype, are necessary for preserving self-tolerance and immunological homeostasis. In an earlier report, CD4+ CD25+ FOXp3+ Tregs remarkably repaired experimental lung damage in an animal model, supporting anti-inflammatory function. Tregs were also found in ARDS patients’ alveolar fluid, indicating a cell-mediated immunotherapy alternative to ARDS that may be adaptable.^45-49^

Corticosteroids were the first immunomodulatory treatment for ARDS, as reported in 1967. Despite being categorized by Ashbaugh et al^1^ as having “Therapeutic trials of doubtful utility,” corticosteroids have long been popular because of their ability to reduce inflammation, which is thought to be the cause of ARDS. To a large extent, the unintended anti-inflammatory effect of corticosteroids is the substantial reduction in lung inflammation and shortening of ventilation duration in ARDS, with variable impact upon mortality rates.^50^ Hereafter, subsequent research must confirm these results and provide more information on the type, timing, and duration-related challenges of corticosteroids. While corticosteroid studies generally show improvement in lung injury, mortality has not been continually enhanced.^51^ Pre-clinical lung injury models indicate a hyperinflammatory condition that requires pleiotropic immunosuppression using corticosteroids. Nevertheless, refractory lung injury accounts for a small proportion of mortality in adult ARDS or PARDS; multisystem organ failure and neurologic impairment are more frequently implicated in fatalities than hypoxemia.^15,51^ As a result, owing to the dissociation of lung injury from several causes of ARDS death, corticosteroids may not consistently reduce mortality in studies despite reducing lung injury. Alternately, it is probable that any favorable benefits on lung injury are neutralized by the unintended adverse impact due to corticosteroids like neuromuscular weakness or elevated risk of subsequent infections.^52^

Steroids may be beneficial in PARDS. However, patients may have various adverse side effects owing to their wide-ranging mode of action, which affects numerous processes. The primary adverse effects of this treatment were gastrointestinal bleeding, immunosuppression that worsened the existing infection, metabolic disorders including hyperglycemia, retention of sodium chloride and water, potassium loss, hypertension, behavioral symptoms like sleeplessness, psychosis, and delirium, and hampered growth in patients who were continuously given steroid treatment. The aforementioned consequences of steroid treatment could be clinically significant in severely unwell patients with PARDS.

## An overview of the evidences supporting or opposing the use of steroids in PARDS

### 1. Supporting evidence for steroid usage

**Figure 2:**
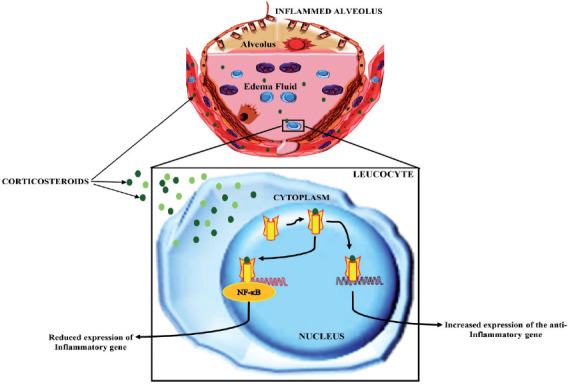
- Routes by which corticosteroids in ARDS inhibit inflammation and interaction between NF-kB and activated corticosteroid receptor

Owing to the anti-inflammatory properties of steroids, they are among the most frequently administered drugs in hospitals, and their use has been extended to treat other diseases over time. Glucocorticoids are involved in both humoral and cellular systems, which have diverse anti-inflammatory effects. The mode of action of corticosteroids is to suppress or prevent inflammation, which can occur due to various stimuli such as immunological, radiative, mechanical, chemical, and viral stimuli. Corticosteroids interacts with certain intracellular receptors in the target tissues thereby, controlling the activity of the desired gene. Alteration in target gene expression leads to the generation of differential protein levels in tissues. Most corticosteroid-mediated effects on the genome take time to manifest because of the late onset of gene expression and protein biosynthesis.^53^ An overview of the mode of action of corticosteroids is depicted in Figure 2.

#### 1.1 Steroid treatment lowers the duration of ventilator stay and mortality risk

A meta-analysis was performed with 1371 adult patients from 9 different studies that determined the benefits of administering glucocorticoids in ARDS patients.^54^ The pooled analysis results clearly show that administration of glucocorticoids resulted in a lower death rate (relative risk [RR], 0.83; 95% confidence interval (CI) 0.74–0.93; *p*=0.01; I^2^=37), and sequential trial analysis supported the statistical significance.^54^ Additionally, there is evidence that glucocorticoids may notably increase the number of days without a ventilator on day 28 (mean deviation 3.66 days, 95% CI 2.64–4.68; *p*<0.01) and increase oxygenation (standardized mean difference 4.17; 95% CI 2.32–6.02; *p*=0.01).^54^ Additionally, glucocorticoid administration did not result in enhancing the risk of developing newer infections (RR 0.84; 95% CI 0.70–1.01; *p*=0.07) or hyperglycemia (RR 1.11; 95% CI 0.99–1.23; *p*=0.06).^54^ Administering glucocorticoids may lower the fatalities in ARDS patients. In addition to the standard treatment for ARDS, glucocorticoids could be suggested; however, more studies are required to decide the ideal dosage and period of steroid therapy.

In another study, a subsequent small trial in the case of persistent (minimum 7 days) ARDS revealed the potential benefit of low-dose but sustained treatment with methylprednisolone (beginning with 2 mg/kg/day dose and then eventual weaning), which piqued interest after a brief high-dose trial of methylprednisolone (120 mg/kg/day) failed to show benefit in ARDS. A more extensive experiment that could not confirm these results suggests that starting methylprednisolone post-14 days after ARDS onset could have deleterious effect. In all studies, there was improvement in breathing duration, indicating that methylprednisolone improved functioning of the lungs. Nevertheless, a large study showed an increased rate of neuromuscular weakening and re-administration of methylprednisolone, and no positive benefit of steroid treatment was observed compared to placebo in 60 days. A recent open-label study of dexamethasone for acute ARDS treatment reported a marked reduction in mortality and the time a patient spends on ventilator.^55^

#### 1.2 Steroid treatment improves oxygen saturation index in patents

The study performed by Mitting et al^56^ on a cohort of 78 PARDS patients who had received methylprednisolone over 5 years (2011–2016) showed a >20% increase in oxygen saturation index in 60% of the patients. Regarding steroid treatment, 59% of the patients showed a response. Both the respondents and non-responders shared the same initial characteristics. In “responders,” there was a statistically significant increase in survival to PICU release (74% versus 41% OR 4.14 (1.57–10.87) *p*=0.004). Response of the PARDS patients to the use of steroids was observed to be an independent predictor of patient’s survival to PICU discharge on the multivariate analysis, including probable confounders (*p*=0.002).

In contrast to responders, non-responders died sooner post-administration of steroids (*p*=0.003). In this cohort of PARDS patients, oxygen saturation index (OSI) improvement was reported in 60% of the patients when low-dose methylprednisolone medication was started. Baseline traits could not be used to distinguish between responders and non-responders. After starting methylprednisolone, a 20% increase in OSI was observed; OSI is an independent predictor of the patient survival. However, more prospective studies are necessary to ascertain the effectiveness of the steroid therapy.^56^

#### 1.3 Steroid treatment reduces levels of inflammatory cytokines

De Luca et al^57^ revealed a connection between high inflammatory cytokine levels and ARDS in infant bronchoalveolar lavage fluid. Tumor necrosis factor–α and secretory phospholipase A2 concentrations above normal were linked to severe disease state. The effectiveness of reducing inflammatory cytokines in treating PARDS was considered in this study. Since corticosteroids have been shown to suppress the generation of inflammatory cytokines, corticosteroids may be crucial in regulating the pathological inflammation associated with ARDS.

### 2. Evidence against the use of steroids

Steroids have not consistently improved patient-centered outcomes, despite lowering the severity of lung illness in ARDS. Ventilator-induced lung injury is a key confounding factor that has been observed in many trials. The administration of steroids may have been beneficial because it reduced the continuing inflammation triggered by harmful ventilator settings. If steroids are helpful, they will probably be valid for specific groups that belong to the broad category of ARDS. Most pediatric patients expected to benefit from steroids also have ARDS along with asthma or reactive airway disease of prematurity; however, these patients are understudied. Although steroids would intuitively seem beneficial, and there is a critical need for therapeutic options to enhance PARDS outcomes, the evidence from RCT and meta-analyses studies is still inconclusive. The available research has several shortcomings that prevent it from being used to make firm recommendations. Arguments opposing the steroid administration in ARDS are due to the absence of conclusive data about the benefits and concerns about adverse effects, like hyperglycemia, hypertension, immunosuppression, and neuromuscular weakness, especially when steroids are taken throughout a lengthy ventilatory course.

There is a shortage of information whether corticosteroids should be administered in children with ARDS. Corticosteroids use in PARDS has not yet been the subject of significant randomized controlled research. Although studies have documented varied corticosteroid dose regimens in different PARDS cases, the number of such reports is too small to draw any firm conclusion.

#### 2.1 Corticosteroids have a non-significant effect on Intensive Care Unit mortality

The PALICC panel, in 2015, advised against the routine administration of corticosteroids to children suffering from PARDS.^58^ The advice was entirely based on the case series and case reports, as there were no RCT studies that were performed in children when the recommendations were made.^59-62^ Although some outcomes, such as the number of days without a ventilator and improved cardiopulmonary physiology, showed positive results, steroids are not currently advised to be routinely used for treating adult ARDS patients.

Recent studies have reported that corticosteroid treatment did not improve the survival of children with ARDS. Drago et al^63^ performed the first randomized controlled trials to treat children with PARDS using corticosteroids. Thirty-five patients with PARDS were randomly administered a placebo or methylprednisolone, where methylprednisolone was given as a 2 mg/kg bolus, 1 mg/kg/day for 7 days, and then the treatment was then slowly weaned off during the next seven days. On day eight and during ICU transfer, patients who were administered steroids had better PaO_2_/FiO_2_ (arterial oxygen partial pressure/fractional inspired oxygen) ratio and lower oxygen demand. However, there were no substantial changes in the period of hospitalization, length of ICU stay, ventilator-free days, or mortality.

A meta-analysis of the existing data on steroids in adult ARDS was performed by Ruan et al.^64^ Ten cohort studies and 18 RCTs were included for the meta-analysis. Seven hundred twenty-five participants with moderate heterogeneity were analyzed in the RCTs. They demonstrated that corticosteroids did not affect 60-day mortality; however, they might have had a statistically insignificant impact on ICU mortality. In cohort trials with 749 participants, there was no impact of corticosteroids on ICU mortality rates, however, a non-significant rise in 60-day mortality was observed. The subgroup analysis revealed that steroid response differed depending on the cause of ARDS and the time of start of steroid treatment. Patients with influenza-related ARDS who received steroid treatment exhibited greater death rate when the etiology was considered.^65,66^ In another study undertaken by Tongyoo et al^67^ 197 adult patients having sepsis-associated ARDS were administered either hydrocortisone 50 mg or a placebo after every six hours for a period of seven days. Regarding PaO_2_/FiO_2_ and lung injury ratings, hydrocortisone was linked to a significant improvement in pulmonary physiology; however, it had no discernible effect on the patient’s survival or mechanical ventilation duration.

#### 2.2 Hyperglycemia

Unlike adults, kids have much lower baseline risks of developing metabolic conditions like hyperglycemia. However, children receiving higher doses of steroids have an excessive rate of hyperglycemia than children receiving lower doses of steroids.^68^ No such studies have been carried out to date showing the effect of steroids in causing hyperglycemia during the treatment of PARDS. However, various studies have reported hyperglycemia as a side-effect during the treatment of ARDS in adults. The immune system is negatively impacted by hyperglycemia because it compromises T-lymphocyte apoptosis, immunoglobulin, and granulocyte function. Having high blood sugar encourages the growth of bacterial and fungal diseases. It has been shown in numerous research that hyperglycemia increases the likelihood and severity of infection. Studies have demonstrated that pediatric patients with acute lymphocytic leukemia (ALL) and steroid-induced diabetes have an increased risk of infection, and that diabetes negatively impacts the survivability of children with ALL.^69^

#### 2.3 Infection

Administration of heavy and chronic doses of corticosteroids induces hyperglycemia, increasing the possibility of infection. However, the corticosteroid is sufficient to create the chance of infection by suppressing the immune system of patients by either blocking CD4+ T cells or suppressing the transcription of cytokines.^70^ Chronic doses of corticosteroids impact the number of natural killer cells in the patients leading to an immune-compromised state. Furthermore, a reduction in ROS generation and elevated levels of pro-inflammatory cytokines (such as IL-6 and TNF-α), together with a rise in apoptosis and reduction in T- and B-cells, possibly contributing in elevating infection risk.^71^

In conclusion, as per the PALICC guidelines, steroids are not recommended as a part of the standard PARDS care. Although steroid therapy for pediatric and adult ARDS is not entirely out of the question, the sensible use of glucocorticoids for ARDS cases is based on the reasonable assumption that the individual belongs to a specific group of steroid responders.

## Current guidelines on the use of steroids in PARDS

As previously stated, the 2015 PALICC recommendations do not include corticosteroids as a standard course of treatment for PARDS. They met the “strong agreement” requirement and strongly advised against using steroids in patients with PARDS.^58^

Application of steroids (similar dosage of methylprednisolone 1–2 mg/kg/day) for adult ARDS patients is advised by 2 Asian ARDS guidelines. This suggestion is not based on decreased mortality but because of the favorable impact of low-dose steroids administration during the treatment of early ARDS, which results in diminished hypoxemia along with shorter duration of mechanical breathing. The Scandinavian clinical practice guidelines of 2016 advise that routine corticosteroid treatment in individuals having ARDS should be avoided, regardless of the dose or duration. At the same time, there are no specific European recommendations. Guidelines for the corticosteroid therapy in adults with ARDS were released by the Faculty of Intensive Care Medicine (FICM) and the Intensive Care Society (ICS) in 2018, however, the recommendations were only intended for scientific studies and not for normal steroid usage in hospitals. The committee suggested conducting a multicenter RCT to assess corticosteroid use in individuals with ARDS. There is no published information on the practices currently used in Latin America.^68-71^

In conclusion, corticosteroids are frequently used in pediatric therapy to treat respiratory illnesses owing to their anti-inflammatory properties. However, the choice of providing steroids for severe respiratory illness should be supported by data on their effectiveness. The treatment with corticosteroids in patients with PARDS remains a critical clinical issue as there is insufficient information regarding its use in children. The results from adult research are inconsistent, with some trials demonstrating a definite advantage and others contradicting this evidence. This could be explained by the vast variations in dosage schedules, selection of patients, and the overall outcomes of the trials. As a result of the diverse clinical types of PARDS, steroids are not expected to provide a uniform therapeutic response, making general recommendations for steroid use challenging.
